# Preparation and Characterization of Highly Aligned Carbon Nanotubes/Polyacrylonitrile Composite Nanofibers

**DOI:** 10.3390/polym9010001

**Published:** 2017-01-03

**Authors:** Yanhua Song, Zhaoyang Sun, Lan Xu, Zhongbiao Shao

**Affiliations:** National Engineering Laboratory for Modern Silk, College of Textile and Engineering, Soochow University, 199 Ren-ai Road, Suzhou 215123, China; syanhua2015@163.com (Y.S.); zhaoyangsun1991@163.com (Z.S.); sdszb2015@163.com (Z.S.)

**Keywords:** carbon nanotubes, highly aligned, composite nanofibers, electrospinning

## Abstract

In the electrospinning process, a modified parallel electrode method (MPEM), conducted by placing a positively charged ring between the needle and the parallel electrode collector, was used to fabricate highly aligned carbon nanotubes/polyacrylonitrile (CNTs/PAN) composite nanofibers. Characterizations of the samples—such as morphology, the degree of alignment, and mechanical and conductive properties—were investigated by a combination of scanning electron microscopy (SEM), transmission electron microscopy (TEM), universal testing machine, high-resistance meter, and other methods. The results showed the MPEM could improve the alignment and uniformity of electrospun CNTs/PAN composite nanofibers, and enhance their mechanical and conductive properties. This meant the successful preparation of highly aligned CNT-reinforced PAN nanofibers with enhanced physical properties, suggesting their potential application in appliances and communication areas.

## 1. Introduction

Polymer nanocomposites with nanoparticles show amazing mechanical, electrical, and thermal properties due to the interaction between the polymer matrixes and nanoparticles [1,2,3]. Carbon nanotubes (CNTs), which have excellent mechanical, physical, and chemical properties—such as good stiffness, high strength, and electrical conductivity—have attracted a great deal of scientific interests in the area of appliances, communication, and biomedical related applications [4,5,6,7,8]. However, a number of challenges must be overcome before producing a homogeneous dispersion of CNTs in a polymer matrix, including processing methods for fabricating CNTs/polymer composites.

Electrospinning (ES) is an efficient technique for the preparation of CNTs/polymer composite nanofibers. The mechanical properties of the electrospun nanofibers are expected to be enhanced through stretching and making composites. There are some reports concerning the preparation of electrospun CNTs/polymer nanofibers [9,10,11,12,13]. However, these electrospun composite nanofibers generally show randomly oriented nonwoven structures and weak alignment. The randomly oriented composite nanofibers lead to low molecular orientation, and as a result, materials with low mechanical properties are obtained [14,15]. The relatively low mechanical strength could ultimately limit the practical use of composite nanofibers. It is desirable to generate aligned composite nanofibers to broaden the application areas, such as electrochemical sensors, reinforcements, optoelectronic devices, and so on.

**Figure 1:**
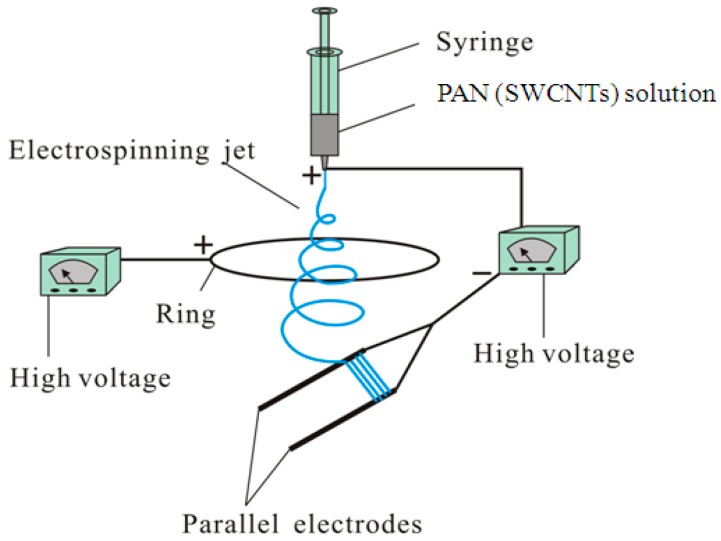
Schematic of the modified parallel electrode method (MPEM) apparatus.

Some techniques have been proposed to obtain aligned nanofibers [16,17,18,19,20,21,22,23]. Previously, we presented a modified parallel electrode method (MPEM) by placing a positively charged ring between the needle and the parallel electrode collector to fabricate highly aligned electrospun nanofibers for a long spinning time [24]. Figure 1 shows the schematic presentation of the modified ES apparatus. In this work, the experimental and theoretical analyses were carried out to study the mechanical mechanism of the MPEM, and the effects of the applied ring on quality of products were systematically investigated to obtain the optimal parameters. The results showed the MPEM could decrease the nanofiber diameter, enhance the diameter distribution, and improve the nanofiber alignment.

Recently, using the ES technique, many functional nanomaterials have been developed using various polymers, such as poly(vinylidene fluoride) (PVDF) [25], poly(methyl methacrylate) (PMMA) [26,27], polylactic acid (PLA) [28], and poly-*N*-vinylpyrrolidone (PVP) [29,30]. Polyacrylonitrile (PAN), a well-known polymer with good stability and mechanical properties, has been widely used in producing electrospun nanofibers due to their excellent characteristics, such as spinnability, environmentally benign nature, and commercial availability [31].

Based on the above considerations, highly aligned PAN composite nanofibers containing single-walled carbon nanotubes (SWCNTs) with concentrations ranging from 0 to 2 wt % were prepared directly by the MPEM technology without any additional postprocessing. Characterizations of the SWCNTs/PAN composite nanofibers—such as morphology, the degree of alignment, and mechanical and conductive properties—were studied by means of scanning electron microscopy (SEM), transmission electron microscopy (TEM), universal testing machine, high-resistance meter, and other methods. The results showed the MPEM could improve the uniformity of diameter distribution and the degree of alignment of electrospun SWCNTs/PAN composite nanofibers. In addition, mechanical and conductive properties of the composite nanofibers were enhanced. As a result, for the CNT-reinforced PAN nanofibers, strong interfacial adhesion, uniform dispersion, and high alignment were more crucial factors for improving the physical properties of the composite nanofibers.

## 2. Experimental

### 2.1. Materials

PAN powder, the biodegradable polymer used in this study, with a molecular weight of 150,000 g/mol, was supplied by Beijing Lark Branch Co. Ltd. (Beijing, China). *N,N*-dimethylformamide (DMF) (analytical reagent) was purchased from Shanghai Chemical Reagent Co. Ltd. (Shanghai, China). The SWCNTs were purchased from Shanghai Aladdin Biochemical Technology Co. Ltd. (Shanghai, China) (purity: ≥98%, ACOOH content: 2 wt %, inner diameter: <2 nm, length: 5–30 μm). All materials were used without any further purification.

### 2.2. Preparation of Spinning Solution

All concentration measurements were done in weight per weight (*w*/*w*). Initially, different weights of SWCNTs were dispersed in the DMF by using an ultrasonic cleaner (SL-5200DT, Nanjing Shunliu Instrument Co. Ltd., Nanjing, China) for 4 h at 25 ± 2 °C (room temperature). Then, the electrospinning solution was prepared by dissolving 8 wt % of PAN in SWCNTs/DMF solution under magnetic stirring for 6 h at room temperature (25 ± 2 °C) until it became homogeneous. The calculated SWCNTs’ concentrations for each component of the various samples were 0, 0.5, 1, 1.5, and 2 wt % (mass ratios to PAN).

### 2.3. Fabrication of Highly Aligned SWCNTs/PAN Composite Nanofibers

Highly aligned PAN composite nanofibers containing SWCNTs with concentrations ranging from 0 to 2 wt % were prepared directly by the MPEM. The schematic of the MPEM apparatus is represented in Figure 1. The apparatus consisted of a syringe, a needle, a parallel electrode collector, a flow meter, and two variable DC high-voltage power generators (0–30 kV, DW-P303-1ACF0, Tianjin DongWen high-voltage power generator Co., Ltd., Tianjin, China). The needle was connected to the positive terminal of the first power generator, and the parallel auxiliary electrodes as the collection target were connected to the negative terminal of the same power generator. The voltage supplied by the power generator was designated as the spinning voltage. The positive terminal of the second power generator was connected to a copper ring, and the voltage provided was referred to as the ring voltage [24]. The ring was 21 cm in diameter.

ES experiments were carried out at room temperature (25 ± 2 °C) at a relative humidity of 50%. The prepared SWCNTs/PAN/DMF solution was dropped into a 10 mL syringe. The flow rate was 0.5 mL/h. The applied spinning voltage was 15 kV and the applied ring voltage was +5 kV. The distance from the tip of the needle to the parallel auxiliary electrodes was 18 cm, the distance from the ring to the parallel electrode collector was 5 cm, and the gap between two parallel auxiliary electrodes was 4 cm.

### 2.4. Measurements and Characterizations

#### 2.4.1. Scanning Electron Microscopy (SEM)

The morphologies of highly aligned SWCNTs/PAN composite nanofibers were examined by a scanning electron microscopy (SEM, Hitachi S-4800, Tokyo, Japan) at an acceleration voltage of 3 kV. All samples were dried at room temperature, and then sputter-coated with gold by an IB-3 (Eiko, Tokyo, Japan) for 10 min. The matrix morphology and fibrous diameter characterization were carried out using ImageJ software (National Institute of Mental Health, Bethesda, MD, USA).

#### 2.4.2. Transmission Electron Microscopy (TEM)

The distribution of SWCNTs in the composite nanofibers was characterized by a transmission electron microscopy (TEM, Tecnai G2 F20 S-Twin, Hillsboro, OR, USA) operating at 200 kV. The samples were prepared by placing the carbon-coated copper grids on the collector and directly depositing a thin layer of electrospun nanofibers onto the copper grids. TEM images were subsequently obtained by passing a beam of electrons through the copper grids containing the composite nanofibers at a high voltage of 200 kV, a dark current of 10.57 μA, and an emission current of 64 μA.

#### 2.4.3. Fourier-Transform Infrared (FTIR) Spectroscopy and Raman Spectroscopy

The PAN structure and its interactions with the SWCNTs were investigated through FTIR spectroscopy (Nicolet5700, Thermo Nicolet Company, Madison, WI, USA). For each measurement, each spectrum was obtained by the performance of 32 scans with the wavenumber ranging from 400 to 4000 cm^−1^ and a resolution of 4 cm^−1^. Raman spectra for the pure SWCNTs and the SWCNTs/PAN composite nanofibers were obtained on a Micro-Raman Spectrometer (LabRAM XploRA, HORIBA JY, Paris, France) at 532 nm laser excitation (KIT-532-25) with edge filter and laser filter. The laser power density was kept as 25 mW. Energy range of Raman spectrum was from 200 to 4000 cm^−1^, all the integration time of the Raman tests was 5 s, and the accumulation was 10 times.

#### 2.4.4. X-ray Diffraction (XRD)

X-ray diffraction (XRD) analyses were performed to elucidate the crystalline structure of powdered SWCNTs, PAN, and SWCNTs/PAN composites using Philips X’Pert-Pro MPD (PANalytical, Almelo & Eindhoven, The Netherlands) with a 3 kW ceramic tube as the X-ray source (Cu-Kα) and an X’Celerator detector. The TTK sample stage was set horizontally. The reflection peak positions were calibrated with silicon powder (2*θ* > 15°) and silver behenate (2*θ* < 10°). Cu-Kα radiation was used with diffraction angle 2*θ* range of 5°–60° at 40 kV and 40 mA, and the diffraction patterns were collected at a scanning rate of 2°/min.

#### 2.4.5. High-Resistance Meter and Universal Testing Machine

The surface resistance was measured by high-resistance meter (ZC36, Shanghai, China) at room temperature and ambient condition. The tensile strength and elongation-at-break values of PAN and SWCNTs/PAN composite nanofiber membranes were investigated by a universal electromechanical test machine Instron-3365 (Instron Corporation, Boston, MA, USA). The electromechanical test machine is well known for its ability to perform a wide range of quasi-static tension and compression tests at test speeds up to 40 ipm (1000 mm/min). All samples were 50 mm × 10 mm rectangle membranes. All measurements were carried out at room temperature. The measurement was repeated three times.

## 3. Results and Discussion

### 3.1. Morphological Characterization of Highly Aligned SWCNTs/PAN Composite Nanofibers (SEM)

**Figure 2:**
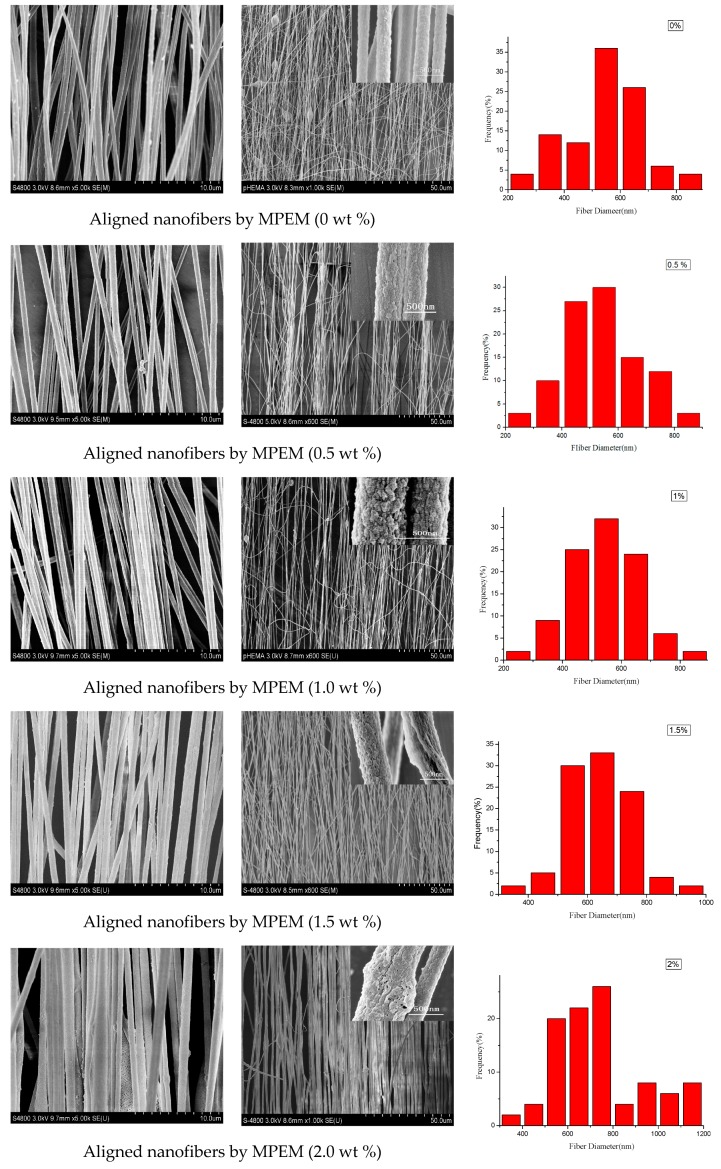

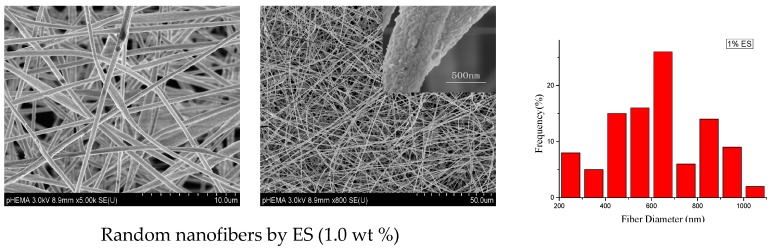
Scanning electron microscopy (SEM) pictures of single-walled carbon nanotubes/polyacrylonitrile (SWCNTs/PAN) composite nanofibers with different SWCNT concentrations. The right most figures are the according diameter distribution. ES: electrospinning.

**Figure 3:**
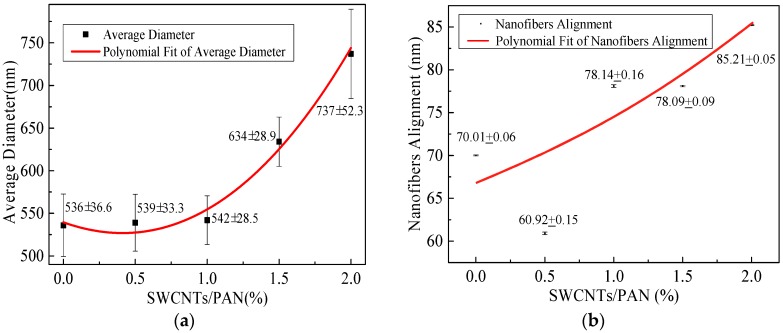
Average diameter and nanofiber alignment of composite nanofibers with the different SWCNTs concentrations. (**a**) Average diameter; (**b**) Nanofiber alignment.

The morphologies of SWCNTs/PAN composite nanofibers were carried out by SEM. To determine the diameter distribution of nanofibers, 50 SEM images were chosen for diameter distribution analysis using ImageJ software. Figure 2 illustrates SEM images of the composite nanofibers with the different SWCNT concentrations, and the rightmost figures are the according nanofiber diameter distribution. It can be seen that the MPEM decreases the composite nanofiber diameter, enhances the diameter distribution, and improves the composite nanofiber alignment. With the increase of the SWCNT content, the diameters of the composite nanofibers increased and their surface became rough, which could be related to the aggregation of SWCNTs and the bundling phenomenon during the MPEM process. The relationship between the content of the SWCNTs and the average diameters of composite nanofibers is shown, respectively, in Figure 3a and Table 1. In Table 1, the standard deviation values (*σ*) are high due to estimation of diameters of fibers based on observed sample data. Therefore, a confidence interval gave an estimated range of values, which was likely to include unknown diameters of fibers. The estimated range was calculated from a given set of sample data [32].

Figure 3b displays the degree of alignment of the composite nanofibers. In this research, the angle (*θ*) between the long axis of the nanofibers and its expected direction (the vectors of parallel electric field) was used as the parameter to quantify the alignment. The degree of nanofiber alignment was defined as the ratio of the number of nanofibers, whose *θ* is between −6° and 6°, to the total number of nanofibers. To determine the degree of nanofiber alignment, several SEM images were captured from each sample. Finally, 50 images were chosen for alignment analysis. With the concentration of SWCNTs increased from 0 to 2 wt %, the average diameters of composite nanofibers were 536, 539, 542, 634, and 737 nm and the degrees of their alignment were 70%, 60.9%, 78.1%, 78.1%, and 85.2%. In this study, the viscosity and electrical conductivity of the spinning solution increased with the increase of SWCNTs concentration from 0 to 2 wt %. With the increase of the viscosity, the polymer chains resist electric field stretching, and the bending instability of jets can be suppressed. As a result, both the diameter and degree of alignment of composite nanofibers increased [1]. In addition, with the increase of the electrical conductivity, the surface charges of the electrospun jet increased, and the resultant force produced by the ring increased, according to our previous work [24]. This would increase the kinetic energy of the moving jet, accelerate the downward movement of the jet, and shrink the radius of whipping circle [24]. As a result, the stability condition and the composite nanofiber alignment were improved and the diameter became much smaller. Therefore, the diameter first remained nearly constant and then increased, and the degree of alignment increased gradually, considering the combined effects of the viscosity and electrical conductivity.

### 3.2. Characterization of SWCNT Distribution in the Composite Nanofibers (TEM)

**Figure 4:**
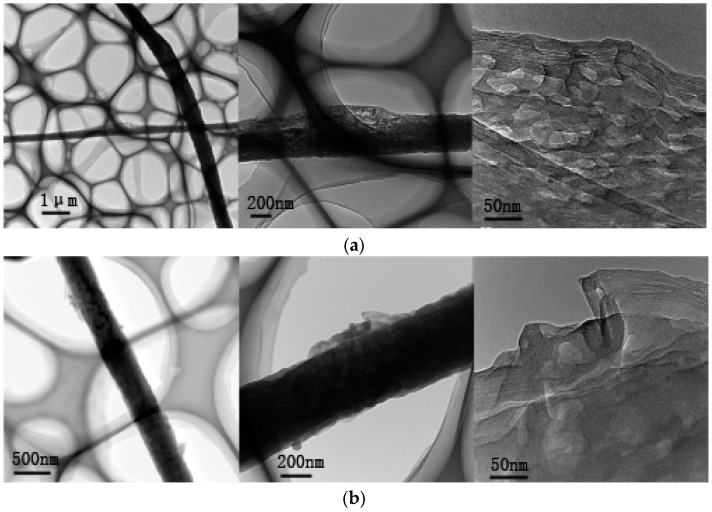
Transmission electron microscopy (TEM) photographs of the composite nanofibers with different SWCNT content. (**a**) 1 wt %; (**b**) 2 wt %.

The distribution of SWCNTs in the highly aligned SWCNTs/PAN composite nanofibers were researched by TEM. Figure 4 displays the TEM photographs of the composite nanofibers with SWCNTs contents of 1 and 2 wt %, respectively. The figure illustrates the SWCNTs were embedded in the polymer matrix and almost aligned along the composite nanofiber axis. When the content of SWCNTs increased to 2 wt %, the surface of the composite nanofibers became rough, which could be related to the aggregation of SWCNTs and the bundling phenomenon occurring during the MPEM process.

### 3.3. FTIR and Raman Spectra Analysis

**Figure 5:**
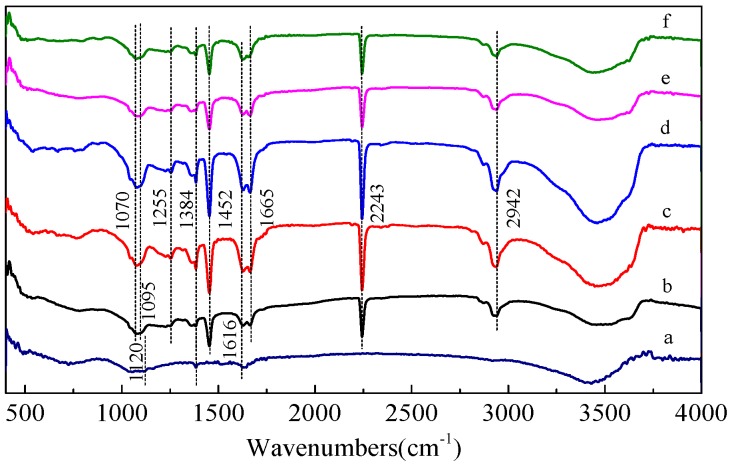
Fourier-transform infrared (FTIR) spectra of (**a**) SWCNTs; (**b**) PAN nanofiber; and corresponding composite nanofibers with the different SWCNT concentrations of (**c**) 0.5 wt %; (**d**) 1 wt %; (**e**) 1.5 wt %; (**f**) 2 wt %.

Figure 5 shows the FTIR spectra for pure SWCNTs, PAN nanofibers, and corresponding composite nanofibers containing SWCNTs. The intermolecular interactions between PAN and SWCNTs were investigated by FTIR analysis, which showed minor changes in the spectra of the pure PAN nanofiber upon addition of SWCNTs. One of these subtle modifications was observed at the 3300–3600 cm^−1^ region, indicating that the π electrons present in SWCNTs interact with the hydrogen (free and bonded) attached to the nitrogen in the urethane bond, thus changing the shape of the band. Moreover, the spectra showed sharp peaks at 1616 cm^−1^ (Figure 5a,c–f), which was due to the C=C stretch mode in the SWCNTs.

**Figure 6:**
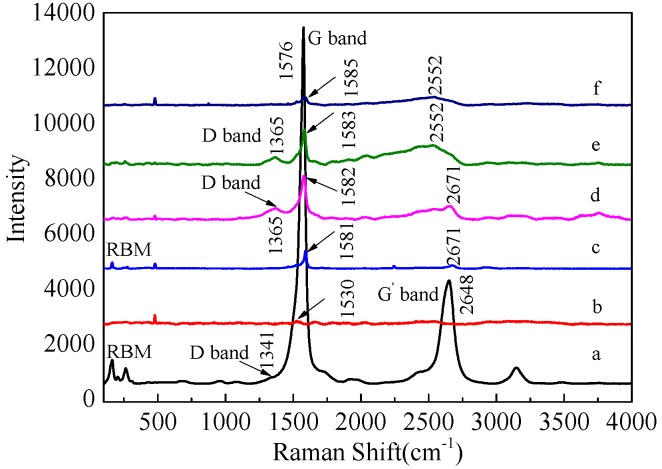
Raman spectra of (**a**) SWCNTs; (**b**) PAN nanofiber; and corresponding composite nanofibers with the different SWCNT concentrations of (**c**) 0.5 wt %; (**d**) 1 wt %; (**e**) 1.5 wt %; (**f**) 2 wt %.

Raman spectra of the PAN nanofibers, pristine SWCNTs, and corresponding composite nanofibers containing SWCNTs are presented in Figure 6, respectively. It has been well known that the ratio of G-band to D-band depends on both the degree of graphitization and the alignment of graphitic planes [33]. Figure 6a shows G-band (1576 cm^−1^), D-band (1341 cm^−1^), and G’-band (2648 cm^−1^) for the samples, indicating their effective carbonization. Moreover, in the region from 350 to 120 cm^−1^, SWCNTs yielded a series of bands that had been assigned to the radius breathing mode (RBM). From the spectra (Figure 6c–f), it was confirmed that the SWCNT/PAN composites showed shifts of several Raman bands of SWCNTs, such as the G-band showing a small shift from 1576 to 1585 cm^−1^, the D-band shifting from 1341 to 1365 cm^−1^, and the G’-band shifting from 2648 to 2671 cm^−1^. Moreover, the *I*_G_/*I*_D_ value of composite nanofibers increased upon increasing the concentration of SWCNTs. However, the spectra for SWCNT/PAN composites at different concentrations did not show the polymer peaks because these were “masked” by the presence of the SWCNTs.

### 3.4. XRD Spectra

**Figure 7:**
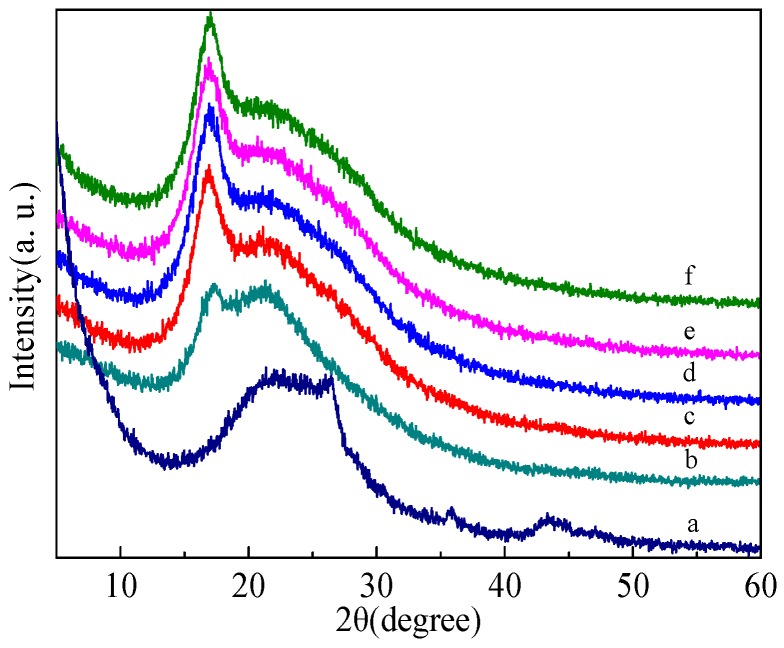
X-ray diffraction (XRD) spectra of (**a**) SWCNTs; (**b**) PAN nanofibers; and corresponding composite nanofibers with the different SWCNT concentrations of (**c**) 0.5 wt %; (**d**) 1 wt %; (**e**) 1.5 wt %; (**f**) 2 wt %.

In order to elucidate the crystalline structure of SMWNTs, PAN, and SMWNT/PAN composites, their XRD patterns with distinctive crystalline peaks are shown in Figure 7. Figure 7b is the XRD spectra for the electrospun PAN nanofibers. A strong peak was observed at 2*θ* = 17° and a weak peak at 2*θ* = 21.5°. The strong peak can be assigned as (200) crystal planes of PAN [34]. The crystallization nature of SWCNTs is shown in Figure 7a. The peak at 2*θ* = 26.5° indicated the typical signal of CNTs or graphite structures. This peak was associated with the (002) diffraction of the hexagonal graphite structure in the carbon materials. Figure 7c–f shows the XRD spectra of the composite nanofibers with the different SWCNT concentrations. It could be observed that the SWCNT/PAN nanocomposites exhibited an obvious sharp diffraction peak of at 2*θ* = 17° and a less intense peak at 2*θ* = 21.5°. The principal peak of graphene structure at 26.5° did not appear, probably because of the high ratio of PAN composite with respect to SWCNTs [1,35]. The XRD results illustrated that there was no new crystalline phase in the composite nanofibers, and SWCNTs and PAN still retained their crystalline structures.

In addition, these XRD patterns contained both sharp as well as defused bands. Sharp bands corresponded to crystalline orderly regions and defused bands corresponded to amorphous regions. The crystallinity was calculated by separating intensities due to amorphous and crystalline phase on diffraction phase. Computer-aided curve-resolving technique was used to separate crystalline and amorphous phases of the diffracted graph. After separation, the total area of the diffracted pattern was divided into crystalline ($A_{c}$) and amorphous components ($A_{a}$). It was not difficult to obtain $A_{c}$ and $A_{a}$ using a curve-fitting Gaussian technique. The crystallinity $X_{c}$ could be calculated from $A_{c}$ and $A_{a}$ using the relation $X_{c}=\frac{A_{c}}{A_{c}+A_{a}}\times 100\%$, and the results are illustrated in Table 2. Table 2 shows the crystallinities of SWCNT/PAN composite nanofibers were higher than pure PAN nanofiber, and remained nearly constant. It was possible that the addition of SWCNTs induced the recrystallization of the polymer molecular chains in the amorphous region and incomplete crystalline region in the electrospinning process.

### 3.5. Mechanical Properties Analysis (Universal Testing Machine)

**Figure 8:**
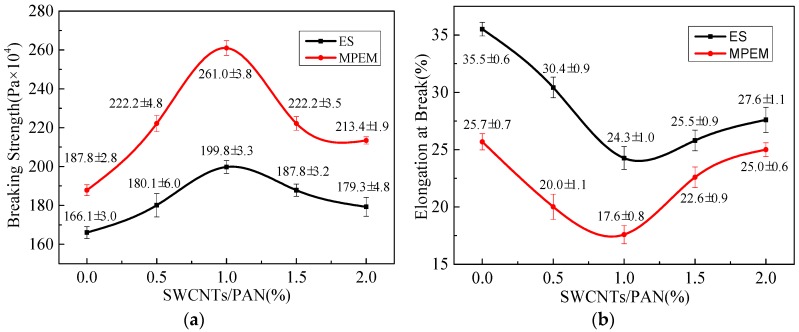
Mechanical properties of random and aligned SWCNTs/PAN nanofiber membranes with the different contents of SWCNTs. (**a**) Tensile strength (MPa); (**b**) elongation at break (%).

The mechanical properties, such as tensile strength and elongation-at-break, of random and aligned SWCNTs/PAN nanofiber membranes with the different SWCNT concentrations are shown in Figure 8. It was seen that the tensile strength of nanofibers firstly increased and then decreased, and the elongation of nanofibers firstly decreased and then increased. When the content of SWCNTs was 1 wt %, the tensile strength of SWCNTs/PAN nanofibers reached the maximum value, which could be related to the well-dispersed and aligned SWCNTs in the polymer nanofibers under the content below 1 wt %. In addition, the alignment degree of nanofibers had a profound effect on the mechanical properties. For aligned nanofiber membranes, the combination of adding SWCNTs and the ordered array made the tensile strength of SWCNTs/PAN composite nanofiber membranes increase significantly. This implied that the incorporation of SWCNTs in polymer nanofiber structure generally could give rise to stronger but fewer flexible nanofiber layers. Moreover, the increase in tensile strength and drop in elongation-at-break has also been reported [1].

### 3.6. Surface Resistance (High-Resistance Meter)

The surface resistances of random and aligned SWCNTs/PAN nanofiber membranes with the different SWCNT concentrations were measured, respectively. As shown in Table 3, with the increase of SWCNT content from 0.5 to 1.5 wt %, the surface resistances of random and aligned SWCNTs/PAN nanofiber membranes varied from approximately 1 × 10^15^ to 1 × 10^10^ Ω/square. It could be seen that the alignment degree of nanofibers had a profound effect on the electrical conductivity, and the combination of adding SWCNTs and the ordered array made the electrical conductivity of the composite nanofiber membranes increase significantly.

## 4. Conclusions

In this work, a modified parallel electrode method (MPEM), carried out by placing a positively charged ring between the needle and the parallel electrode collector, was successfully used to fabricate highly aligned SWCNTs/PAN composite nanofibers with the different SWCNT concentrations. The properties—such as morphology, the degree of alignment, crystallinity, mechanical properties, and electrical conductivity—of highly aligned SWCNTs/PAN composite nanofibers with SWCNT concentrations, which ranged from 0 to 2 wt %, were investigated. The results showed the properties of PAN nanofibers were improved by adding SWCNTs.

SEM and TEM photographs showed that with the increase of SWCNT contents, the diameters of the composite nanofibers increased and their surface became rough moreover, the SWCNTs were embedded in the polymer matrix and almost aligned along the composite nanofiber axis. In addition, the measurement of mechanical properties and surface resistances revealed that the tensile strength and electrical conductivity of aligned composite nanofibers were obviously improved over that of random composite nanofibers. The results showed that the MPEM could improve the alignment of electrospun CNTs/PAN composite nanofibers, and enhance their mechanical and conductive properties.

## Figures and Tables

**Table 1:** The relationship between the content of the SWCNTs and the average diameters of composite nanofibers.

Method	SWCNTs concentration (wt %)	Average diameter ($\bar{D}$) (nm)	Standard deviation (*σ*) (nm)	Confidence interval (nm)
MPEM	0	536	132.8	±36.6
MPEM	0.5	539	120.7	±33.3
MPEM	1	542	103.7	±28.5
MPEM	1.5	634	104.8	±28.9
MPEM	2	737	189.6	±52.3
ES	1	635	184.4	±36.1

**Table 2:** The crystallinity of SWCNTs, PAN nanofibers, and corresponding composite nanofibers with the different SWCNT concentrations.

Sample	Crystallinity (%)
SWCNTs	85.69
PAN nanofibers	49.3
SWCNTs/PAN nanofibers with 0.5 wt % SWCNTs	55.62
SWCNTs/PAN nanofibers with 1 wt % SWCNTs	55.76
SWCNTs/PAN nanofibers with 1.5 wt % SWCNTs	55.76
SWCNTs/PAN nanofibers with 2 wt % SWCNTs	55.79

**Table 3:** Surface resistances of random and aligned SWCNTs/PAN nanofiber membranes with the different content of SWCNTs.

SWCNTs concentration (wt %)	Surface resistance (Ω)	
	MPEM	ES
0	1.7 × 10^14^	6.0 × 10^15^
0.5	2.5 × 10^12^	2.4 × 10^15^
1	1.5 × 10^11^	4.5 × 10^13^
1.5	9.2 × 10^9^	2.3 × 10^12^
